# O12.7. RISK OF PSYCHOSIS IN OFFSPRING OF PARENTS WITH A HISTORY OF HOMELESSNESS DURING CHILDHOOD AND ADOLESCENCE: A NATIONWIDE, REGISTER-BASED, COHORT STUDY

**DOI:** 10.1093/schbul/sby015.275

**Published:** 2018-04-01

**Authors:** Sandra Feodor Nilsson, Thomas Munk Laursen, Carsten Hjorthøj, Anne Thorup, Merete Nordentoft

**Affiliations:** 1 University of Copenhagen; 2 Aarhus University, The Lundbeck Foundation Initiative for Integrative Psychiatric Research (iPSYCH); 3 Copenhagen University Hospital, Mental Health Center Copenhagen; 4 Research Unit, Child and Adolescent Mental Health Center, Capital Region of Denmark and The Lundbeck Foundation Initiative for Integrative Psychiatric Research (iPSYCH); 5 Mental Health Centre Copenhagen, The Lundbeck Foundation Initiative for Integrative Psychiatric Research (iPSYCH)

## Abstract

**Background:**

Children and adolescents with deprived backgrounds have high rates of psychiatric problems. Parental and social factors are regarded crucial for children’s healthy and positive development but whether children’s risk of psychosis in an early age is associated with parental social marginalisation is unknown. We aimed to analyse the association between mothers’ and fathers’ history of homelessness and the offspring’s risk of psychiatric disorders during childhood and adolescence with attention to the specific child and adolescent psychiatric diagnosis: psychosis, as well as to the combined effect of mother’s and father’s schizophrenia and bipolar disorder and homelessness experiences according to the child’s risk of any psychiatric disorder.

**Methods:**

We conducted a nationwide, register-based cohort study of 1,072,882 children aged 0−16 years living or being born in Denmark between Jan 1, 1999 and Dec 31, 2015. Parental homelessness was the primary exposure and offspring’s risk of psychosis and other psychiatric disorders the outcome. We analysed the association by survival analysis using Poisson regression and incidence rate ratios (IRRs), adjusted for year and offspring characteristics, and additionally adjusted for parental factors (age at offspring’s birth and parental psychiatric disorders).

**Results:**

In total, 17,238 (2%) offspring had either one or two parents with a history of homelessness, and 56330 (5%) offspring were diagnosed with any psychiatric diagnosis during the study period. Of these, 850 (1.5%) had a diagnosis of psychosis before their 16th birthday. The incidence rate of any psychiatric disorder was 28.2 cases per 1000 person-years (20.7–38.4) in offspring with at least one parent with a history of homelessness and a mother with a schizophrenia or bipolar disorder, compared with 18.3 cases per 1000 person-years (16.8–20.0) in those whose parents had no history of homelessness.

The IRR of psychosis in offspring born to a mother with a history of homelessness was 3.1 (95% CI 1.9–5.0) compared with those whose parents had no history of homelessness. A similar risk was found if both parents had a history of homelessness (IRR 5.4, 95% CI 2.7-1.9), whereas no association was found when only the father had experiences of homelessness. Also after full adjustment including parental psychiatric disorders, an increased risk of psychosis was found in offspring if the mother (IRR 1.8, 95% CI 1.1–3.0) or both parents (IRR 2.9, 95% CI 1.4–5.9) had a history of homelessness. Highest risk was found for attachment disorder when both parents had a history of homelessness (IRR 32.5, 95% CI 24.6−42.9) and substance use disorder when only the mother experienced homelessness (6.9, 95% CI 4.9−9.7). In offspring whose mother had a history of homelessness and a psychiatric disorder, 36% (95% CI 27%−45%) had received a psychiatric diagnosis themselves by the age of 15. If the mother or father had a schizophrenia or bipolar disorder, homelessness experiences did not add further to the increased risk of any psychiatric disorder in offspring.

**Discussion:**

Parental homelessness was strongly associated with an increased risk of psychosis and several other severe psychiatric disorders in offspring during childhood and adolescence. These findings have important implications for public health and policy because they suggest a need for improvement in the support of socially marginalised families to help prevent psychiatric illness in offspring. However, our findings also suggested that the risk of any psychiatric disorder in offspring associated with parental homelessness depended on the parental psychiatric diagnoses.

